# Supplementary material for: Effects of Whole-Body Adenylyl Cyclase 5 (Adcy5) Deficiency on Systemic Insulin Sensitivity and Adipose Tissue
Source: Int J Mol Sci. 2021 Apr 21;22(9):4353. doi: 10.3390/ijms22094353 (PMC8122634; doi:10.3390/ijms22094353)
Supplement: Supplementary file 1 [file ijms-22-04353-s001.zip › ijms-1182523-supplementary/supplementary/210326 ijms-1118569_SupMat.pdf]

International Journal of *Molecular Sciences*

MDPI

Supplementary

Effects of Whole-Body Adenylyl Cyclase 5 (*Adcy5*) Deficiency on Systemic Insulin Sensitivity and Adipose Tissue

Sebastian Dommel [1], Anne Hoffmann [2], Claudia Berger [1], Matthias Kern [2], Nora Klöting [1,2], Aimo Kannt [3,4,5,†] and Matthias Blüher [1,2,†,*]

[1] Medical Department III—Endocrinology, Nephrology, Rheumatology, Medical Center, University of Leipzig, 04103 Leipzig, Germany; sebastian.dommel@medizin.uni-leipzig.de (S.D.); claudia.berger@medizin.uni-leipzig.de (C.B.); nora.kloeting@medizin.uni-leipzig.de (N.K.)

[2] Helmholtz Institute for Metabolic, Obesity and Vascular Research (HI-MAG) of the Helmholtz Zentrum München at the University of Leipzig and University Hospital Leipzig, 04103 Leipzig, Germany; anne.hoffmann@helmholtz-muenchen.de (A.H.); matthias.kern@helmholtz-muenchen.de (M.K.)

[3] Fraunhofer Institute for Translational Medicine and Pharmacology ITMP, 60596 Frankfurt am Main, Germany; aimo.kannt@itmp.fraunhofer.de

[4] Experimental Pharmacology, Medical Faculty Mannheim, University of Heidelberg, 68167 Mannheim, Germany

[5] Sanofi Diabetes Research and Development, 60596 Frankfurt am Main, Germany

* Correspondence: bluma@medizin.uni-leipzig.de; Tel.: +49-341-9715984

† These authors contributed equally to this work.

Supplemental Tables

Table S1. Primer pairs used for PCR and qRT-PCR

Gene	forward (3' - 5')	reverse (3' - 5')	product size [bp]	
Adcy5-WT	AGTTCCCGTCGGACAAACTG	AGCACGGCCATGATAAGGAT	202	PCR
Adcy5-KO	ATCAGCAGCCTCTGTTCCAC	AGCACGGCCATGATAAGGAT	152	
36b4	ACTGGTCTAGGACCCGAGAAG	TCAATGGTGCCTCTGGAGATT	77	qRT-PCR
Adcy5	TGAACCAGAGCAGTCTCACCAT	GCGCCGCGTGGAAAG	75	

Table S2. Genes differentially expressed between *Adcy5*$^{-/-}$ and Ctrl eWAT of mice on CD.

	log fold change	average expression	t	p value	adjusted p value	B	gene symbol	gene name
	-2.1350	9.9576	-9.5430	0.0000	0.0002	8.2296	Mid1	midline 1
	-1.2712	9.8360	-4.7226	0.0002	0.0666	0.8772	C4a	complement component 4A (Rodgers blood group)
	-1.2594	6.8913	-5.0437	0.0001	0.0522	1.4808	Frzb	frizzled-related protein
	-1.1919	8.1372	-3.0935	0.0066	0.2112	-2.2818	Cybrd1	cytochrome b reductase 1
	-1.1650	6.9036	-3.0256	0.0076	0.2206	-2.4114	Cdh6	cadherin 6
	-1.1649	7.7974	-3.3615	0.0037	0.1808	-1.7647	Trdn	triadin
	-1.0919	6.3408	-3.3050	0.0042	0.1843	-1.8743	Retnlg	resistin like gamma
	-1.0884	11.1019	-4.3140	0.0005	0.0874	0.0934	C4b	complement component 4B (Chido blood group)
	-1.0775	5.9808	-3.2652	0.0045	0.1901	-1.9512	Rimklb	ribosomal modification protein rimK-like family member B
	-1.0567	10.9694	-3.5775	0.0023	0.1634	-1.3437	Thbs2	thrombospondin 2
	-1.0453	10.4473	-3.7806	0.0015	0.1402	-0.9464	Inmt	indolethylamine N-methyltransferase
	1.0144	9.7029	4.5752	0.0003	0.0722	0.5963	Mir7653	microRNA 7653
	1.0144	9.7029	4.5752	0.0003	0.0722	0.5963	Tlcd1	TLC domain containing 1
	1.0268	8.9910	3.8166	0.0014	0.1388	-0.8760	Egln3	egl-9 family hypoxia-inducible factor 3
	1.0391	6.8885	5.3045	0.0001	0.0444	1.9611	Bpifb2	BPI fold containing family B, member 2
female	1.0462	5.2977	3.0891	0.0066	0.2112	-2.2901	Galnt3	polypeptide N-acetylgalactosaminyltransferase 3
	1.0508	9.5822	6.2766	0.0000	0.0196	3.6603	Gpd2	glycerol phosphate dehydrogenase 2, mitochondrial
	1.0510	8.2825	5.0751	0.0001	0.0514	1.5391	Angptl8	angiopoietin-like 8
	1.0562	8.7057	4.8054	0.0002	0.0613	1.0341	Tlcd2	TLC domain containing 2
	1.0604	11.8998	4.6570	0.0002	0.0672	0.7524	Slc25a1	solute carrier family 25 (mitochondrial carrier, citrate transporter), member 1
	1.0643	6.7594	3.1320	0.0060	0.2078	-2.2079	Slc9a4	solute carrier family 9 (sodium/hydrogen exchanger), member 4
	1.0825	7.2708	3.1017	0.0064	0.2112	-2.2660	Paqr5	progestin and adipoQ receptor family member V
	1.0889	10.3534	5.8738	0.0000	0.0245	2.9745	Nsdhl	NAD(P) dependent steroid dehydrogenase-like
	1.0968	8.8846	3.3588	0.0037	0.1808	-1.7699	Sorl1	sortilin-related receptor, LDLR class A repeats-containing
	1.1047	8.2051	3.2458	0.0047	0.1909	-1.9887	Slc14a1	solute carrier family 14 (urea transporter), member 1
	1.1122	7.2845	5.6586	0.0000	0.0301	2.5972	Slc15a5	solute carrier family 15, member 5
	1.1122	8.1601	4.5446	0.0003	0.0733	0.5377	H2-Q10	histocompatibility 2, Q region locus 10
	1.1182	11.4009	5.8725	0.0000	0.0245	2.9722	Acaca	acetyl-Coenzyme A carboxylase alpha
	1.1190	5.1357	3.3586	0.0037	0.1808	-1.7703	Mup5	major urinary protein 5
	1.1196	5.0300	2.9370	0.0092	0.2297	-2.5795	Esrp1	epithelial splicing regulatory protein 1
	1.1223	7.6040	4.8358	0.0002	0.0613	1.0914	Cox8b	cytochrome c oxidase subunit 8B
	1.1236	6.9761	3.5542	0.0024	0.1646	-1.3892	Sidt1	SID1 transmembrane family, member 1

	1.1679	7.5062	3.7879	0.0015	0.1402	-0.9322	Orm2	orosomucoid 2
	1.2060	12.0647	6.1438	0.0000	0.0226	3.4372	Me1	malic enzyme 1, NADP(+)-dependent, cytosolic
	1.2080	5.2496	2.9485	0.0089	0.2276	-2.5577	Tmprss13	transmembrane protease, serine 13
	1.2173	11.5855	2.9670	0.0086	0.2247	-2.5227	Thrsp	thyroid hormone responsive
	1.2173	9.9811	3.1931	0.0053	0.1963	-2.0904	Slc25a10	solute carrier family 25 (mitochondrial carrier, dicarboxylate transporter), member 10
	1.2278	8.1587	3.4684	0.0029	0.1709	-1.5565	Lrmda	leucine rich melanocyte differentiation associated
	1.2404	4.6054	2.9454	0.0090	0.2287	-2.5635	Macc1	metastasis associated in colon cancer 1
	1.3031	7.3486	2.9097	0.0097	0.2342	-2.6311	Grhl3	grainyhead like transcription factor 3
	1.3197	4.1843	3.0958	0.0065	0.2112	-2.2774	Serpinb5	serine (or cysteine) peptidase inhibitor, clade B, member 5
	1.3364	7.7634	3.0887	0.0066	0.2112	-2.2909	Cpt1b	carnitine palmitoyltransferase 1b, muscle
	1.3488	11.8083	6.9307	0.0000	0.0095	4.7157	Mir3076	microRNA 3076
	1.3488	11.8083	6.9307	0.0000	0.0095	4.7157	Tkt	transketolase
	1.3967	11.8683	3.3767	0.0036	0.1795	-1.7350	Fasn	fatty acid synthase
	1.4062	6.5605	3.8735	0.0012	0.1309	-0.7648	Dsp	desmoplakin
	1.4241	6.0365	3.2556	0.0046	0.1909	-1.9699	Cers3	ceramide synthase 3
	1.4548	6.4275	2.9486	0.0089	0.2276	-2.5576	Tspan8	tetraspanin 8
	1.4611	5.3069	3.9119	0.0011	0.1270	-0.6895	Sytl5	synaptotagmin-like 5
	1.5167	8.6081	4.4420	0.0004	0.0787	0.3407	Parm1	prostate androgen-regulated mucin-like protein 1
	1.6143	6.3359	5.7498	0.0000	0.0295	2.7582	Slc2a5	solute carrier family 2 (facilitated glucose transporter), member 5
	1.6502	10.9860	6.1018	0.0000	0.0226	3.3660	Gpd1	glycerol-3-phosphate dehydrogenase 1 (soluble)
	1.6697	6.5850	3.8299	0.0013	0.1374	-0.8501	Tmem184a	transmembrane protein 184a
	1.6720	5.9583	3.4688	0.0029	0.1709	-1.5559	Esrp2	epithelial splicing regulatory protein 2
	1.7240	9.2592	3.3436	0.0038	0.1808	-1.7994	Pnpla3	patatin-like phospholipase domain containing 3
	1.7906	7.7370	2.9710	0.0085	0.2247	-2.5151	Cidea	cell death-inducing DNA fragmentation factor, alpha subunit-like effector A
	1.8410	6.4967	3.1201	0.0062	0.2087	-2.2307	Foxq1	forkhead box Q1
	1.9457	6.1630	3.4392	0.0031	0.1709	-1.6136	Pdzk1ip1	PDZK1 interacting protein 1
	1.9745	7.5593	5.1206	0.0001	0.0495	1.6233	Orm3	orosomucoid 3
	2.0203	7.9754	8.3166	0.0000	0.0011	6.7140	Atp1a3	ATPase, Na+/K+ transporting, alpha 3 polypeptide
	2.0373	5.7460	3.0918	0.0066	0.2112	-2.2851	Cldn8	claudin 8
	2.2891	6.9358	3.2473	0.0047	0.1909	-1.9859	Upk2	uroplakin 2
	2.3718	10.4778	5.5767	0.0000	0.0309	2.4518	Elovl6	ELOVL family member 6, elongation of long chain fatty acids (yeast)
ma	-2.2955	9.9576	-9.2631	0.0000	0.0003	5.3174	Mid1	midline 1
	-1.3969	7.2748	-3.4273	0.0032	0.4642	-1.7319	Fgf13	fibroblast growth factor 13

-1.2474	7.5391	-3.2658	0.0045	0.4730	-1.9929	Cd209d	CD209d antigen
-1.1648	7.2028	-5.0832	0.0001	0.3940	0.8325	Syn3	synapsin III
-1.1431	8.3677	-3.4185	0.0032	0.4642	-1.7462	Alox15	arachidonate 15-lipoxygenase
-1.1081	7.8301	-2.9184	0.0095	0.4779	-2.5512	Cxadr	coxsackie virus and adenovirus receptor
-1.0723	6.6701	-3.6749	0.0019	0.4642	-1.3323	Csprs	component of Sp100-rs
-1.0717	8.3159	-3.2235	0.0050	0.4730	-2.0613	Tnfrsf11b	tumor necrosis factor receptor superfamily, member 11b (osteoprotegerin)
1.0564	6.8235	4.5105	0.0003	0.4296	-0.0169	Sez6l2	seizure related 6 homolog like 2
1.0609	8.1763	3.6228	0.0021	0.4642	-1.4162	Gm3002	alpha-takusan pseudogene
1.1674	7.5081	4.8146	0.0002	0.4296	0.4406	Mir147	microRNA 147
1.1674	7.5081	4.8146	0.0002	0.4296	0.4406	AA467197	expressed sequence AA467197
1.2359	8.7852	3.1791	0.0054	0.4730	-2.1329	Grem2	gremlin 2, DAN family BMP antagonist
1.2946	6.2733	3.1857	0.0054	0.4730	-2.1222	D830030K20Rik	RIKEN cDNA D830030K20 gene
1.3078	8.1580	4.7680	0.0002	0.4296	0.3714	Zyg11a	zyg-11 family member A, cell cycle regulator
1.6634	7.4760	3.1727	0.0055	0.4730	-2.1433	Scd3	stearoyl-coenzyme A desaturase 3
2.4642	10.9302	3.1038	0.0064	0.4730	-2.2542	Mup19	major urinary protein 19

Supplemental Figures

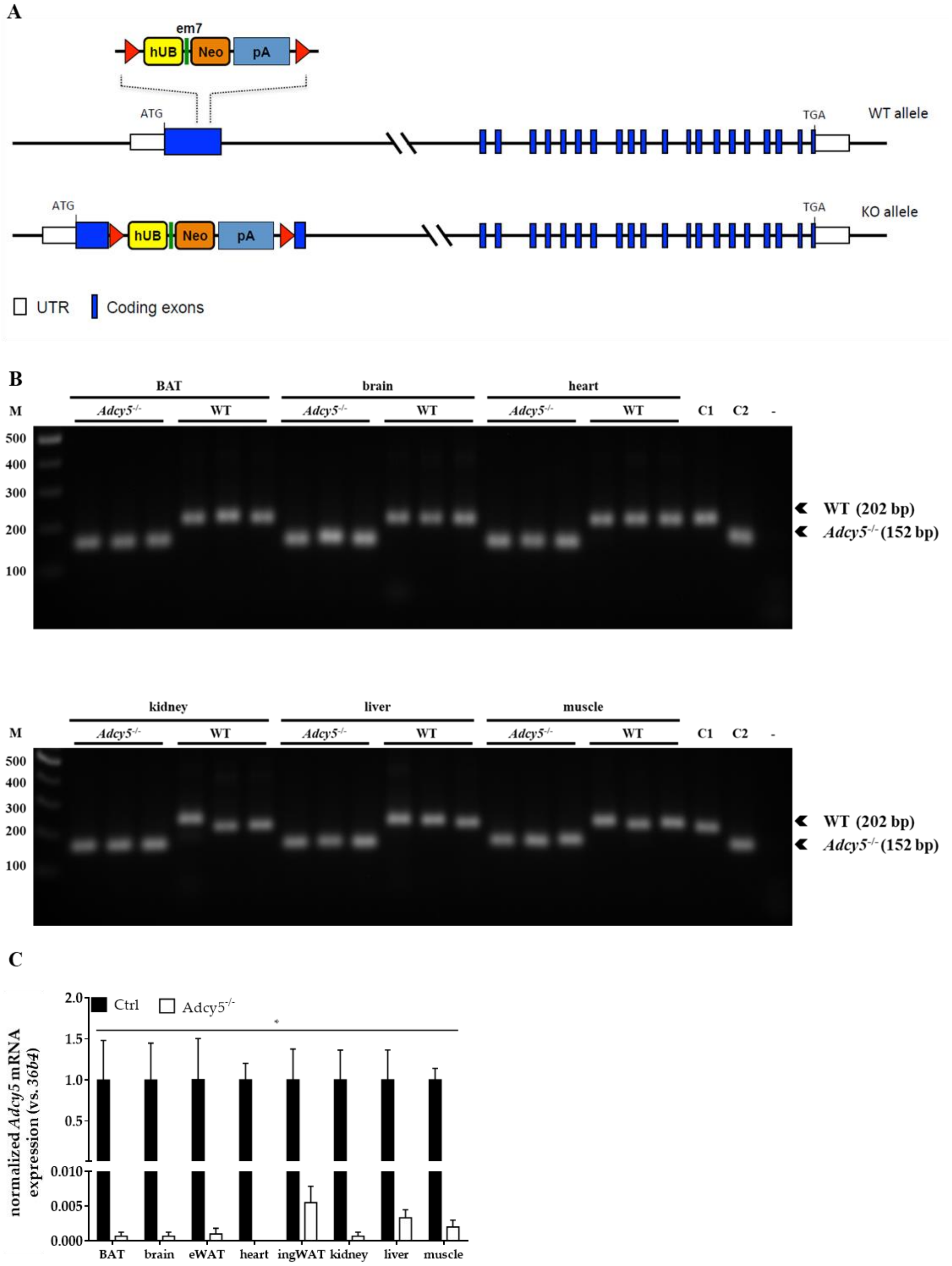

Figure S1. *Adcy5* targeting strategy and knockout validation. *Adcy5*$^{-/-}$ mice were created in analogy to the method described in Okumura *et al.* (18). Successful insertion of loxP-hUBp-em7-Neo-polyA-loxP cassette into exon 1 led to a loss of *Adcy5* expression (*A*). Agarose gel images display characteristic bands for wild type (WT) with a size of 202 bp and *Adcy5*$^{-/-}$ of 152 bp in different tissues (*B*). Relative to WT control *Adyc5* mRNA expression (black: WT; open bars: *Adcy5*$^{-/-}$) is expressed relative to *36b4* in different tissues. In all analyzed tissues, *Adcy5* expression was significantly down regulated (*C*). *n* (C) = 3. Data presented as mean ± SD. * Significantly different between Ctrl and *Adcy5*$^{-/-}$ with $p < 0.05$. hUB – human ubiquitin promoter, em7 – synthetic *E. coli*-derived T7 promoter, Neo – neomycin resistance, pA – polyadenylation site, UTR – untranslated region, WT – wild type, KO – *Adcy5* knockout, BAT – brown adipose tissue, C1 – wild type control, C2 – knockout control, (-) – negative control, M – 100 bp marker, eWAT – epigonadal white adipose tissue, ingWAT – inguinal white adipose tissue, Ctrl – wild type control animals.

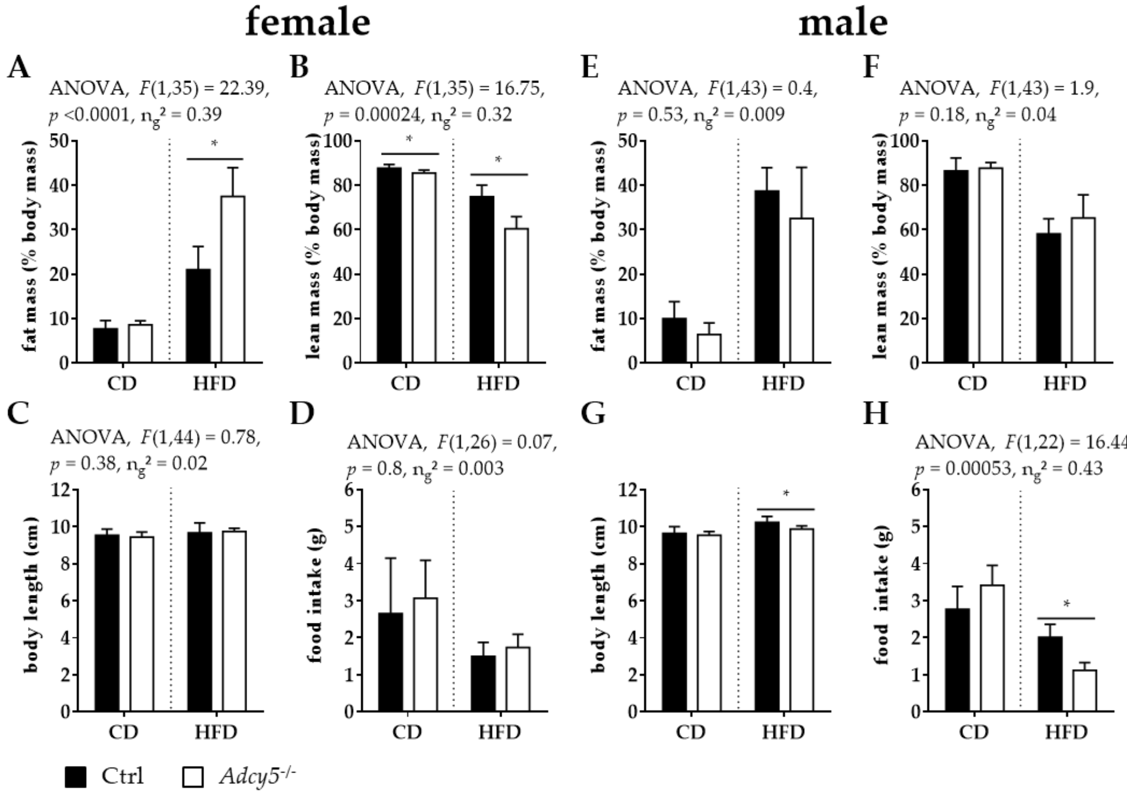

Figure S2. Phenotypic characterization of *Adcy5*$^{-/-}$ versus control (Ctrl) mice. Female *Adcy5*$^{-/-}$ mice (n=11) presented higher relative fat (A) and lower relative lean masses (B) under HFD compared to controls (n=14). Body composition was estimated by EchoMRI700™. Body lengths do not differ between female *Adcy5*$^{-/-}$ and Ctrl mice independently of the diet (C). Food intake (D) was not different between females of both genotypes under CD and HFD. In male mice under CD, relative fat mass (E) was lower in *Adcy5*$^{-/-}$ and relative lean mass (F) mass was higher in *Adcy5*$^{-/-}$ under HFD. Body length (G) was slightly higher in Ctrl compared to *Adcy5*$^{-/-}$ males under HFD. Food intake (H) was not different for males of both genotypes under CD, but lower in *Adcy5*$^{-/-}$ upon HFD. Results are expressed as mean ± SD. Differences within the same diet resulting from the genotype were proved for statistical significance (*) using two-way ANOVA. Post-hoc tests using pairwise t-test were computed for significant interactions only and corrected applying the Bonferroni method. Both CD and HFD data was plotted within the same bar graphs and separated by a dotted line. Number of included animals: n (A and B) = 9 (CD, Ctrl), 5 (CD, *Adcy5*$^{-/-}$). n (C) = 12 (CD per group), 14 (Ctrl, HFD) and 11 (*Adcy5*$^{-/-}$, HFD). n(D) = 7 (CD, Ctrl), 9 (CD, *Adcy5*$^{-/-}$), 8 (Ctrl, HFD) and 7 (*Adcy5*$^{-/-}$, HFD). n (E, F and G): 10 (CD, Ctrl and Adcy5$^{-/-}$), 14 (HFD, Ctrl) and 13 (HFD, *Adcy5*$^{-/-}$).

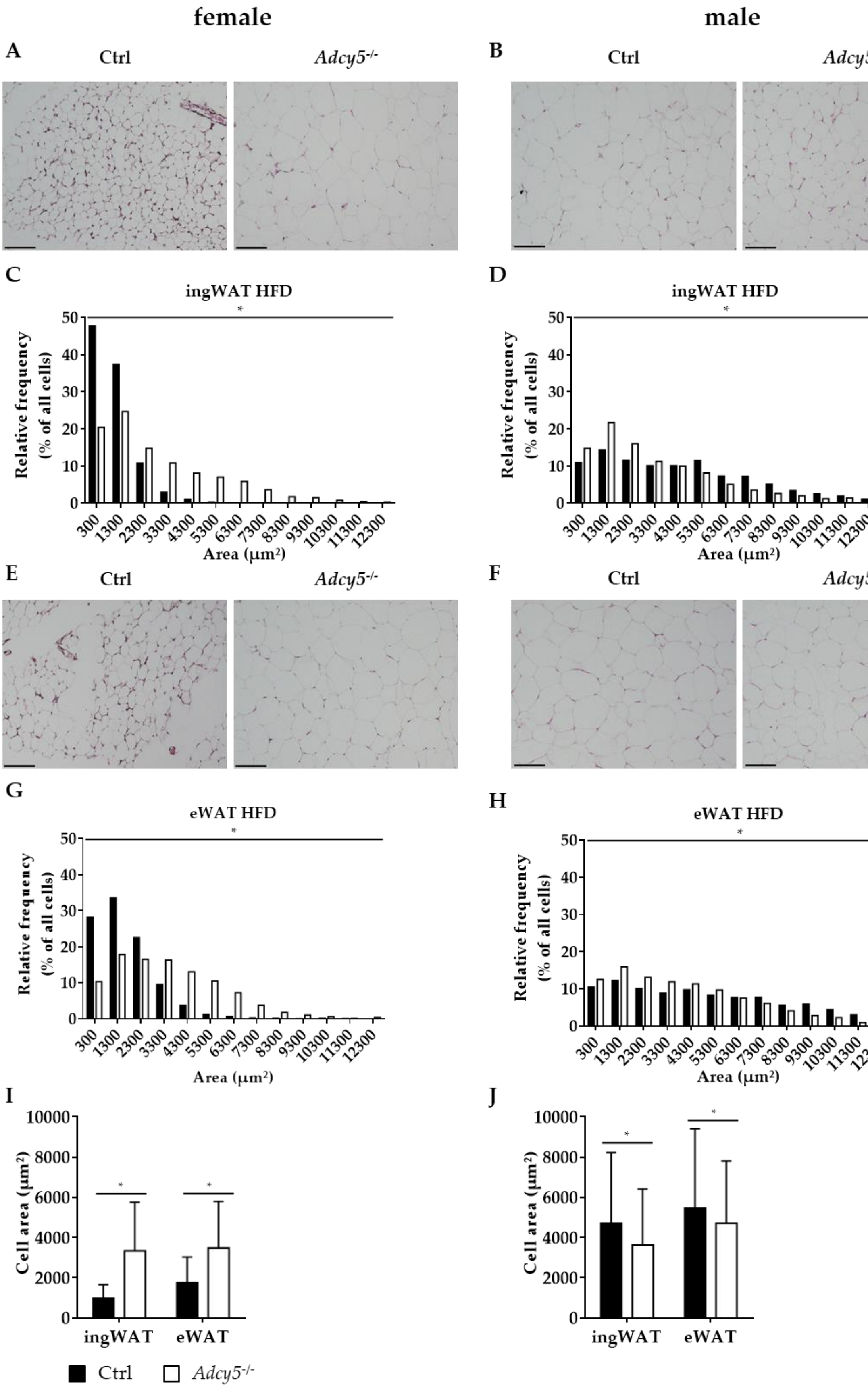

Figure S3. Whole-body *Adcy5* deletion caused smaller adipocytes in white adipose tissue (WAT) only in males after high fat diet (HFD). Representative inguinal WAT (ingWAT) histology sections

of female (*A*) and male (*B*) *Adcy5*$^{-/-}$ and control (Ctrl) mice under HFD. Subfractions of small adipocytes were lower in female (*C*) and not different in male (*D*) *Adcy5*$^{-/-}$ compared to Ctrl mice. (*E*, *F*) Histological slides from epigonadal WAT (eWAT) of female (*E*) and male (*F*) *Adcy5*$^{-/-}$ mice. Subfractions of small adipocytes are lower in female (*G*), but not in male (H) *Adcy5*$^{-/-}$ compared to Ctrl mice. Cell area measurements of female (*I*) and male (*J*) *Adcy5*$^{-/-}$ and Ctrl mice reveal higher mean adipocyte size in both ingWAT and eWAT of females (*I*) and significantly lower mean adipocyte size in male *Adcy5*$^{-/-}$ compared to Ctrl mice (*J*) under HFD. Scale bar = 100 µm. Statistical significance for relative frequencies (C, D, G and H) were calculated by using a Chi^2-test. Data represent mean ± SD. * Significantly different between Ctrl and *Adcy5*$^{-/-}$.
